# A new species of *Apechoneura* Kriechbaumer (Hymenoptera, Ichneumonidae, Labeninae) from Colombia

**DOI:** 10.3897/zookeys.213.3309

**Published:** 2012-08-01

**Authors:** Andrés Fabián Herrera Flórez

**Affiliations:** 1 University of Manitoba, Department of Entomology, 214 Animal Science Bldg, Winnipeg, Manitoba, Canada R3T 2N2

**Keywords:** Ichneumonoidea, Labenini, South America, Neotropics, *nigricornis* species-group, taxonomy

## Abstract

A new species of the ichneumonid subfamily Labeninae, *Apechoneura seminigra* **sp. n.**, is described. Specimens were collected from the Amazon Rainforest of Colombia.

## Introduction

The Labeninae is a subfamily of Ichneumonidae containing approximately 150 described species classified in four tribes and 12 genera. Compared with other subfamilies, this group is quite well-known worldwide (Gauld 2000). The Labenini is a Gondwanan group comprising five genera: *Torquinsha* Gauld & Wahl and *Gauldianus* Lanfranco which are both endemic to Chile; *Labena* Cresson from Australia, Neotropical and Neartic regions; *Certonotus* Kriechbaumer from the Australasian region; and *Apechoneura* which is found in tropical America. Because *Certonotus* shares several autapomorphies with *Apechoneura* (e.g. mesoscutal rugae), *Apechoneura* was considered part of *Certonotus* (Wahl, 1993), but after a phylogenetic study (Gauld and Wahl 2000), *Apechoneura* has been hypothesized as the sister group of the clade embracing *Certonotus* and *Torquinsha*. Some of the autapomorphies of *Apechoneura* are the presence of a highly raised interantennal lamella and a submetapleural carina lacking an anterior lobe, but with a median denticle. *Apechoneura* has 24 described species and 30 estimated (Gauld 2000; Yu et al. 2005). This genus has been found in Bolivia (Mocsary 1905; Townes and Townes 1966), Brazil (Kriechbaumer 1890; Mocsáry 1905; Townes and Townes 1966; Gauld 2000), Chile (Lanfranco 1980), Colombia (Enderlein 1919; Townes and Townes 1966; Gauld 2000; Herrera 2006), Costa Rica (Mocsáry 1905; Townes and Townes 1966; Gauld 2000; Gauld and Wahl 2000), Ecuador (Morley 1913; Townes and Townes 1966; Gauld 2000), Mexico (Hernández-Aguilar et al. 2000; Ruíz-Cancino et al. 2002), Nicaragua (Cameron 1886; Maes 1989), Panama (Cameron 1886; Townes and Townes 1966), Paraguay (Schrottky 1911; Cushman 1920; Townes and Townes 1966), Peru (Carrasco 1972; Mocsáry 1905; Gauld 2000) and Venezuela (Gauld 2000). Costa Rica and Brazil are the countries with the most species (17 and 6 respectively).

Three species of *Apechoneura* are found in Colombia (Yu et al. 2005): *Apechoneura longicauda* Kriechbaumer, 1890 (Enderlein 1919; Gauld 2000; Herrera 2006), *Apechoneura nigricornis* Mocsáry, 1905 (Townes and Townes 1966; Herrera 2006) and *Apechoneura nigritarsis* (Cameron, 1886) (Townes and Townes 1966; Herrera 2006). Another species, *Apechoneura tricoloripes* (Mocsáry, 1905), may also be present in Colombia because it occurs in Costa Rica (Mocsary 1905; Townes and Townes 1966; Gauld 2000), Paraguay (Cushman 1920; Townes and Townes 1966) and Brazil (De Santis et al. 1973). Gauld (2000) divided the genus into six species-groups; the species described here, like *Apechoneura nigricornis*, belongs to the *nigricornis* species-group.

## Material and methods

During an undergraduate project focused on the subfamily Labeninae, 14 of the main entomological collections of Colombia were reviewed (view Appendix). The specimens described here are deposited in the insect collection of the Instituto de Ciencias Naturales (ICN), Universidad Nacional de Colombia, Bogotá, Colombia. The nomenclatural treatment, morphological terminology and taxonomic characters used here follow Gauld (1991, 2000). The species treated in this study were compared with the descriptions made by Brullé (1846), Cameron (1886), Cushman (1920), Gauld (2000), Kriechbaumer (1890), Mocsáry (1905), and Schrottky (1911).

## Systematics

### Genus *Apechoneura* Kriechbaumer, 1890

#### Apechoneura seminigra

Herrera sp. n.

urn:lsid:zoobank.org:act:4CFB6077-71E1-42D0-92FB-77907767B3CC

http://species-id.net/wiki/Apechoneura_seminigra

**Figures 1–4.**
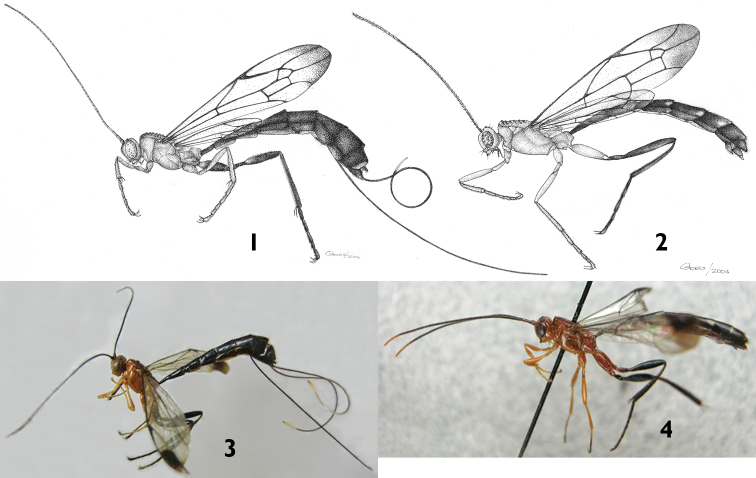
Habitus of *Apechoneura seminigra* sp. n. **1, 3** female, holotype **2, 4** putative male **1, 2** line drawings **3, 4** photographs.

Figures 1 [Fig F2] [Fig F3] 13

##### Material examined.

HOLOTYPE: Female, Colombia, Amazonas: Parque Nacional Natural Amacayacu Caño Mata Matá, 3°41'N, 70°15'W, Malaise trap, Martin Kelsey: 200 m, II-III.1989 (ICN 083474). PARATYPES: 1 female, same data as holotype (ICN 083472); 1 female, same locality, 300 m, 1.III.1988, bosque de tierra firme (ICN 083471).

Non-type material: 1 male, same locality, bosque de várzea (ICN 083473).

##### Diagnosis.

This species can be diagnosed from all other Neotropical *Apechoneura* by the combination of the following: head orange; mesosoma and legs mostly orange (hind leg partly black); metasoma black. Epicnemial carina absent. Metapleuron with a conspicuous sharp lateral denticle. Hind wing with first abscissa of *Cu*1 0.2× as long as *cu-a*.

##### Description.

Female. Fore wing length 15.0 mm.

**Head.** Clypeus almost flat, with a weak transverse ridge near apex; malar space 0.6× as long as basal mandibular width; lower face at narrowest point 0.9× as wide as height from clypeofacial suture to level of insertion of antenna; hypostomal carina joined to occipital carina far from base of mandible; posterior ocellus separated from eye by 1.3–1.5× its own maximum diameter. Antenna with flagellomeres 1 and 2 subequal by length; subapical flagellomere slightly elongate.

**Figures 5–8. F2:**
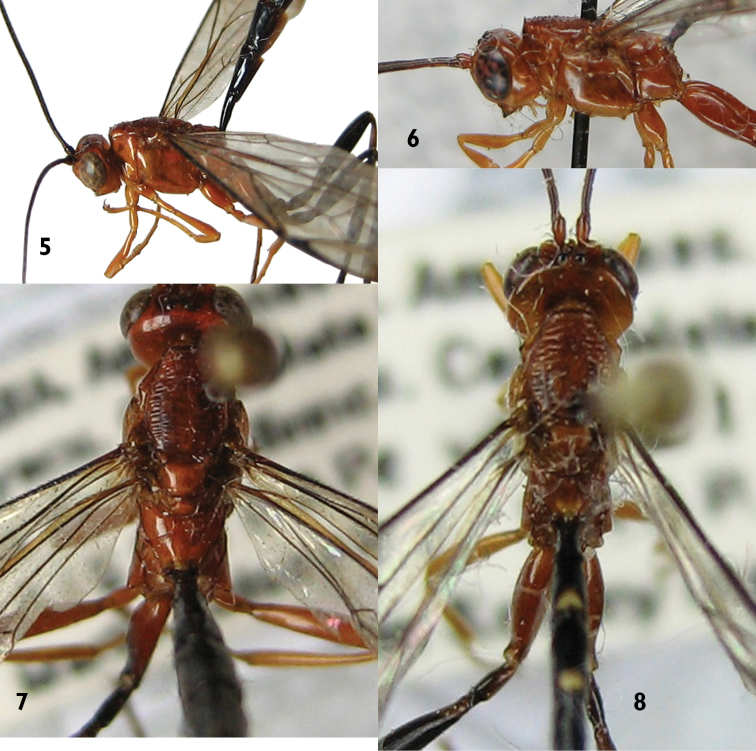
*Apechoneura seminigra* sp. n. **5, 7** female, holotype **6, 8** putative male **5, 6** Head, mesosoma and part of metasoma, lateral view **7, 8** head, mesosoma and part of metasoma, dorsal view.

**Figure 9. F3:**
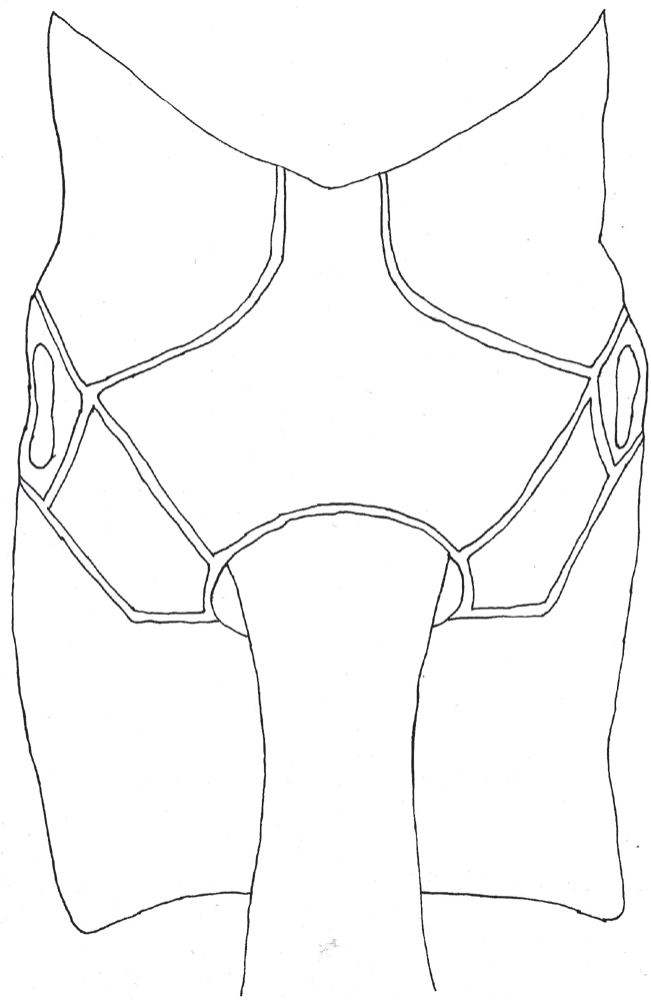
*Apechoneura seminigra* sp. n., female, holotype - propodeum, dorsal view.

**Mesosoma.** Pronotum with upper hind margin swollen, forming a small conical projection; scutoscutellar groove broad and shallow; scutellum with three evident rugae posteriorly; epicnemial carina absent (Figs 1, 3, 5); sternal region of mesothorax smooth and polished; metapleuron with a rather conspicuous sharp lateral projection near posterior end; submetapleural carina narrow with a distinct low median denticle (Fig. 1). Propodeum in profile more or less flat; anterior transverse carina complete laterally, separating area spiracularis from area lateralis, mediodorsally incomplete so area basalis is not enclosed posteriorly; area basalis slightly transverse; lateromedian longitudinal carina not present behind anterior transverse carina (Figs 7, 9).

**Legs.** Fore leg with tibia slightly inflated, tarsus with long hairs on inner surface; mid leg with tibia bearing several stout spines.

**Wings.** (Fig. 12) Fore wing with areolet large, anteriorly narrowly truncate, with 2*m-cu* joining it very slightly basal of middle; second discal cell short, with vein 1*m-cu* about half as long as abscissa of *Cu*1 between *Rs*&*M* and *1m*-*cu*; hind wing with apical abscissa of *Cu*1 joining *cu-a* clearly closer to *M* than to 1*A*; first abscissa of *Cu*1 0.2× as long as *cu*-*a*.

**Metasoma.** Tergite 1 slender, 3.5–4.0× as long as posteriorly broad; sternite 1 short, reaching about 0.3–0.4 of length of tergite, with a median swelling centrally. Tergite 2 1.9–2.3× as long as posteriorly broad, with isolated pubescence; tergite 7 mediodorsally without an indentation posteriorly; tergite 8, in lateral view, tapered to a bluntly rounded apex, without a cornus, and with uniformly scattered pubescence; tergite 9 bearing long pubescence. Ovipositor, at rest extending beyond apex of metasoma by 3.5–3.8× the length of the metatibia.

**Figures 10–13.**
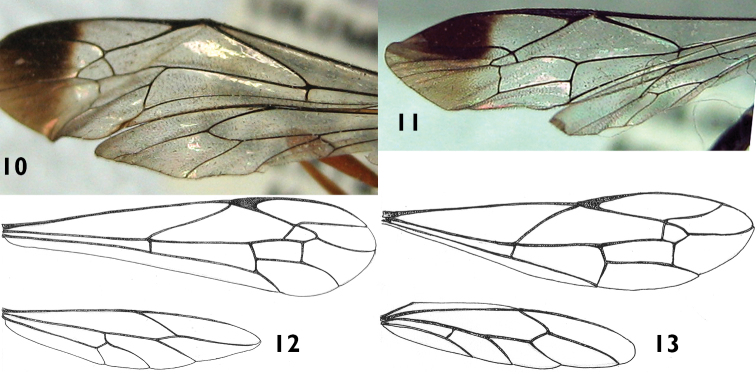
Wings of *Apechoneura seminigra* sp. n. **10, 12** female, holotype; **11, 13** putative male.

**Color.** (Figs 3, 5, 7, 10) Head orange; flagellum predominantly black, two basal flagellomeres ventrally reddish. Mesosoma orange. Fore and mid legs orange; hind leg with coxa orange with a ventro-lateral black spot on the apex of the outer side, trochanter and trochantellus black except for some small orange spots, femur, tibia and tarsus black. Metasoma black, hypopygium centrally orange. Ovipositor sheath black except for subapical whitish wide band. Fore wing hyaline, with a distinctive apical black band; pterostigma black.

**Variation.** The female identified with the code ICN 083471 has the fore wing with the areolet petiolate.

**Putative Male.** Similar to female in structure, but smaller (fore wing length 10.0 mm). Hind wing with apical abscissa of *Cu*1 arising from *M* apical to junction of *M* + *Cu*1 with *cu-a* (Figs 11, 13). Antenna black with apical flagellomeres pale (Fig. 4). Metasoma mostly black, tergites 1–6 with a yellow triangular spot at posterior margin (Figs 4, 8). Metapleuron with denticle smaller and paler than in the female.

##### Specimen condition.

The male exemplar was deteriorated during the drawing process. Its antenna was broken and lost.

##### Etymology.

The species name refers to its color (i.e. metasoma and most part of hind leg black).

##### Remarks.

*Apechoneura seminigra* sp. n., just like *Apechoneura nigricornis*, lacks an epicnemial carina; this characteristic separates them from the rest of the species of the genus. As *Apechoneura nigricornis*, *Apechoneura seminigra* sp. n. possesses a conical projection on the metapleuron and lacks an indentation on tergite 7. However, the metasoma is orange in *Apechoneura nigricornis* and black in *Apechoneura seminigra* sp. n. Also, in the hind wing, the first abscissa of *Cu*1 is 0.4× as long as *cu-a* in *Apechoneura nigricornis* and 0.2 in *Apechoneura seminigra* sp. n. Although these two species are rather similar morphologically, the difference in color pattern makes in this case their separation reliable. Gauld (2000) examined extensive material of *Apechoneura nigricornis* from Costa Rica but also some material from Brazil and Peru, and Herrera (2006) examined one specimen of *Apechoneura nigricornis* collected in Porce (Antioquia, Colombia) in 1998 and no color variation compared to the holotype of this species was found.

##### Comments.

*Apechoneura seminigra* sp. n. is so far only known from Colombia, Amazonian Region, northwest of Leticia. According to Gauld (2000) there is significant sexual dimorphism in *Apechoneura* and the sex association is often difficult, in part because the male specimens are less frequently collected. Despite the differences between the female specimens and the male specimen of *Apechoneura seminigra* sp. n., especially in the hind wing venation, they are tentatively considered here as belonging to the same species, mainly because all the specimens were collected in the same exact locality, in two consecutive years.

## Supplementary Material

XML Treatment for Apechoneura seminigra

## Appendix

List of collections

- Instituto Humboldt (Acronym: **IAvH-E**)

- Insect Collection, Instituto de Ciencias Naturales, Universidad Nacional de Colombia, Bogotá (Acronym: **ICN**)

- Museo de Entomología “Francisco Luis Gallego”, Universidad Nacional, sede Medellín. (Acronym: **UNCM**)

- Instituto de Biología, Universidad de Antioquia, Medellín. (Acronym: **CEUA**)

- Facultad de Agronomía, Universidad Nacional de Colombia; Bogotá (Acronym: **UNAB**)

- Museo de Historia Natural, Pontificia Universidad Javeriana; Bogotá (Acronym: **MUJ**)

- Corporación para Investigaciones Biológicas; Medellín (Acronym: **CIB**)

- Universidad Pedagógica Nacional; Bogotá (Acronym: **UPNC**)

- Edgard Palacio Insect Collection- Colección Personal; Bogotá (Acronym: **EPIC**)

- Museo Universidad La Salle, Bogotá. (Acronym: **U La Salle**)

- Colección Entomológica “Luis María Murillo”, Instituto Colombiano Agropecuario, Tibaitabá. Bogotá (Acronym: **CELM**)

- Museo de Ciencias Naturales, Colegio San José. Medellín, Barrio Boston. (Acronym: **CSJ)**

- Colección entomológica Piedras Blancas (Comfenalco) (Acronym: **CEPB**)

- Colección personal del profesor Oscar E. Ortega M. (Oficina- Unalmed) (Acronym: **OOCP**)
